# The Anesthesiologist's Perspective Regarding Non-intubated Thoracic Surgery: A Scoping Review

**DOI:** 10.3389/fsurg.2022.868287

**Published:** 2022-04-04

**Authors:** Giulio Luca Rosboch, Paraskevas Lyberis, Edoardo Ceraolo, Eleonora Balzani, Martina Cedrone, Federico Piccioni, Enrico Ruffini, Luca Brazzi, Francesco Guerrera

**Affiliations:** ^1^Department of Anesthesia, Intensive Care and Emergency, “Città della Salute e della Scienza di Torino” Hospital, Torino, Italy; ^2^Department of Cardiovascular and Thoracic Surgery, “Città della Salute e della Scienza di Torino” Hospital, Torino, Italy; ^3^Department of Surgical Science, University of Turin, Torino, Italy; ^4^Anesthesia and Intensive Care Unit, General and Specialistic Surgical Department, Arcispedale Santa Maria Nuova, Azienda USL – IRCCS di Reggio Emilia, Reggio Emilia, Italy

**Keywords:** non-intubated thoracic surgery, anesthesia, NITS, VATS, thoracic surgery, regional anesthesia

## Abstract

Non-intubated thoracic surgery (NITS) is a growing practice, alongside minimally invasive thoracic surgery. To date, only a consensus of experts provided opinions on NITS leaving a number of questions unresolved. We then conducted a scoping review to clarify the state of the art regarding NITS. The systematic review of all randomized and non-randomized clinical trials dealing with NITS, based on Pubmed, EMBASE, and Scopus, retrieved 665 articles. After the exclusion of ineligible studies, 53 were assessed examining: study type, Country of origin, surgical procedure, age, body mass index, American Society of Anesthesiologist's physical status, airway management device, conversion to orotracheal intubation and pulmonary complications rates and length of hospital stay. It emerged that NITS is a procedure performed predominantly in Asia, and certain European Countries. In China, NITS is more frequently performed for parenchymal resection surgery, whereas in Europe, it is mainly employed for pleural pathologies. The most commonly used device for airway management is the laryngeal mask. The conversion rate to orotracheal intubation is a~3%. The results of the scoping review seem to suggest that NITS procedures are becoming increasingly popular, but its role needs to be better defined. Further randomized clinical trials are needed to better define the role of the clinical variables possibly impacting on the technique effectiveness.

**Systematic Review Registration:**

https://osf.io/mfvp3/, identifier: 10.17605/OSF.IO/MFVP3.

## Introduction

Parallel to the growth of minimally invasive surgical thoracic techniques, non-intubated thoracic surgery (NITS) has been increasingly used (1, 2). NITS procedures appear to avoid either the adverse effects of mechanical ventilation in patients with already impaired pulmonary functional capacity before surgery, and the residual effects of neuromuscular blockers, providing more rapid recovery of respiratory muscle function and less perioperative morbidity (1, 3).

While NITS technique is becoming increasingly popular, there is still no clarity in how it is defined and performed. For example, the procedure is reported in the literature as non-intubated thoracic surgery, but also referred to as tubeless video-assisted thoracoscopic surgery (VATS) or awake thoracic surgery.

Although an expert consensus recently attempted to clarify and establish the critical points of NITS, some perioperative surgical and anesthesiological evaluation variables remained undefined (4).

In order to better clarify the background of NITS, surgical indications, type of patient to be proposed for the procedure, airway management, postoperative complications, and length of stay (LOS) a scoping review based on a systematic literature review was conducted.

### Protocol and Registration

The study protocol was developed using the Preferred Reporting Items for Systematic reviews and Meta-Analyses extension for Scoping Reviews (PRISMA-ScR) guidelines (5) revised by the members of the thoracic surgery research team of “Città della Salute e della Scienza” university hospital (Turin – Italy). The final protocol was registered prospectively with the Open Science Framework on 26^th^ September 2021 (https://osf.io/mfvp3/).

### Eligibility Criteria

Peer-reviewed articles dealing with NITS with the following characteristics were identified as potentially eligible: (1) randomized controlled trials (RCTs) and non-RCTs (NRCTs); (2) published in English; (3) involving adult participants (>18 years old).

### Information Sources and Search Strategy

Potentially relevant studies were searched through September 2021 in Pubmed, EMBASE and Scopus using the search strategies reported in the Supplementary Materials (Supplementary File 1). The results were exported to EndNote V.X9 (Clarivate Analytics, PA, USA), and the duplicates were automatically removed.

### Studies Evaluation and Selection of Sources of Evidence

The review process was carried out in two steps consisting of evaluating the titles, abstracts, and then full text of all publications identified by our searches for potentially relevant manuscripts. For both levels, four authors worked in pairs (GLR, EC, EB, and MC) and screened the articles with conflicts resolved by consensus and discussion with other reviewers.

### Data Charting Process and Data Items and Synthesis of Results

A planned Excel spreadsheet was developed by reviewers to determine which variables to extract used (study characteristics, patient characteristics, surgical procedures, country, age, number of patients, body mass index (BMI), Forced Expiratory Volume in the 1st second (FEV1), diffusing capacity for carbon monoxide (DLCO), American Society of Anesthesiologists (ASA) physical status classification, intraoperative drugs used, type of anesthesia, type of regional analgesia, bispectral index (BIS) utilization, airway management device, conversion to orotracheal intubation (OTI), conversion to thoracotomy, postoperative pain, postoperative pulmonary complications, and postoperative days of hospitalization). The reviewers independently charted the data in pairs. If not available, any ongoing study was contacted to include unpublished data if applicable. We grouped the studies by the type of study (with or without a comparison group). Where we identified a systematic review, we counted the number of studies included in the review that potentially met our inclusion criteria and noted how many studies had been missed by our search.

## Results

### Selection of Sources of Evidence PRISMA

We followed the Preferred Reporting Items for Systematic reviews and Meta-Analyses extension for Scoping Reviews (PRISMA-ScR) guidelines (5). The systematic literature search performed in September 2021 retrieved 665 results. After deduplication, 283 studies were evaluated. Three hundred eighty-two and 234 were, respectively excluded following the first and second evaluation process bringing the number of studies included in the scoping review to 49. To these, four more articles (6–9) previously reported in systematic reviews were added leading the total number of articles included to 53, all published after 2011.

#### General Characteristics of Included Studies

**Figure 1:**
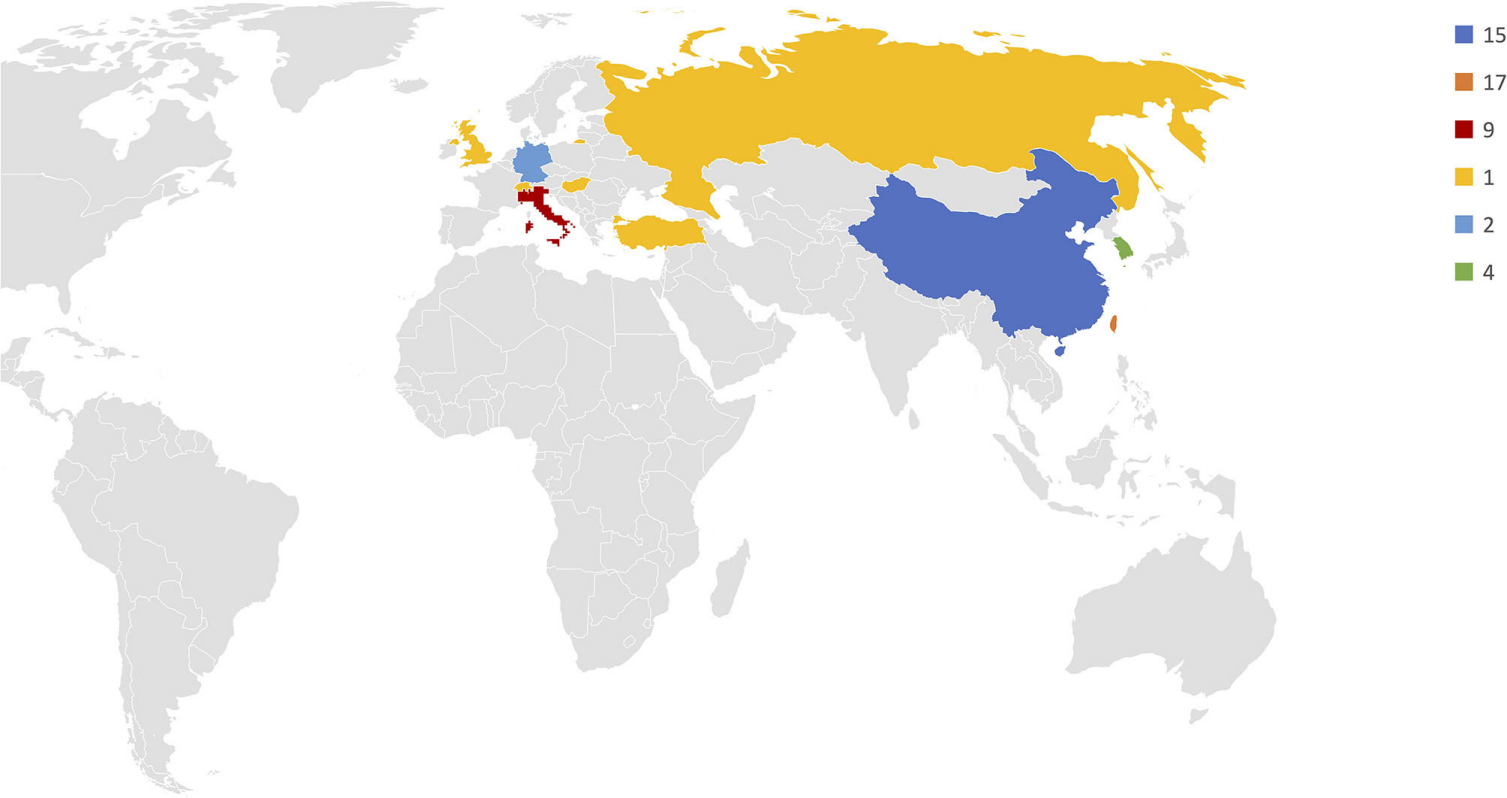
Frequency distribution of studies conducted on NITS.

Among the 53 included studies, 30 were cohort studies comparing NITS and intubated-patient thoracic surgery, whereas 22 were single-cohort studies of patients undergoing NITS. Seventeen were conducted in Taiwan, 15 in China, nine in Italy, four in South Korea, two in Germany and one in Turkey, United Kingdom, Russia, Switzerland, and Hungary (Figure 1). Patients' characteristics are summarized in Table 1.

**Table 1:** Summary table of population characteristics of single-cohort observational studies.

**Author**	**Year**	**Country**	**Type of study**	**Surgical procedure**	**N of patients**	**Age group A**	**Age Group B**	**BMI Group A**	**BMI group B**	**ASA I/II/III/IV (%)**
AlGhamdi et al. (10)	2018	Korea	Retrospective	Lobectomy	62	64.9 ± 10.5	66.1 ± 9.5	23.8 ± 3.2	23.5 ± 2.9	
Ambrogi et al. (11)	2017	Italy	Retrospective	Metastasectomy	58	62 (46–71)	66 (51–73)			
Caviezel et al. (12)	2019	Switzerland	Retrospective	Endoscopic thoracic sympathectomy	20	28.6 (17–46)	28.5 (20–55)	23.6 (17–30.4)	21.8 (19.1–26.3)	
Chen et al. (13)	2011	Taiwan	Retrospective	Lobectomy	30	57.9 ± 10.4	56.5 ± 9.5	24.0 ± 3.2	23.4 ± 3.3	3.3/70/26.7/0
Chen et al. (14)	2016	China	RCT	Endoscopic thoracic sympathectomy	168	23.3 ± 6.8	21.8 ± 6.1			
Chen et al. (15)	2016	China	RCT	Endoscopic thoracic sympathectomy	221	22.9 ± 6.6	21.5 ± 5.4			
Cui et al. (3)	2016	China	Retrospective	Bullectomy	90	24.6 ± 5.6	25.2 ± 11.6	<25	<25	Only ASA I and II
Cui et al. (3)	2016	China	Retrospective	Endoscopic thoracic sympathectomy	89	22.1 ± 7.2	26.5 ± 9.5	<25	<25	Only ASA I and II
Cui et al. (3)	2016	China	Retrospective	Mediastinal tumor resection	91	38.3 ± 11.0	32.7 ± 9.0	<25	<25	Only ASA I and II
Furák et al. (16)	2020	Hungary	Retrospective	Lobectomy	38	64 (63)	63.03 (63)	24.83 ± 3.07	24.31 ± 4.17	
Guerrera et al. (6)	2020	Italy	Prospective	Lung biopsies	94	60.4 ± 2.0	62.1 ± 12.5	26.8 ± 4.8	26.4 ± 4.6	1.5/12.1/80.3 /9.1
Guo et al. (17)	2016	China	Retrospective	Segmentectomy	140	49.10 ± 12.78	56.63 ± 12.70	21.59 ± 2.26	22.49 ± 3.10	25/68.8/6.2/0
Guo et al. (18)	2016	China	Retrospective	Bilateral bullectomy	37	21.9 ± 5.2	26.2 ± 11.4	18.6 ± 2.7	18.9 ± 2.3	Only ASA I and II
Hsiao et al. (19)	2017	Taiwan	Retrospective	Decortication	33	76.4 ± 6.0	76 ± 11.5			
Huang et al. (20)	2020	China	Retrospective	Mediastinal tumor resection	32	63.90 ± 11.76	67.43 ± 14.40	22.01 ± 3.67	23.43 ± 2.25	0/93/7/0
Hwang et al. (21)	2018	Korea	Retrospective	Bullectomy	41					
Irons et al. (22)	2017	United Kingdom	Retrospective	Elective minor VATS procedure	73	54.9 ± 19.3	50.8 ± 19.2	26.2 ± 6.5	25.8 ± 5.8	
Jung et al. (23)	2018	Korea	Retrospective	Bullectomy	183	20.4 ± 7.0	22.9 ± 9.2	19.7 ± 2.5	19.8 ± 2.3	
Ke et al. (24)	2020	Taiwan	Retrospective	Lung resections	160	56.5 ± 16.8	52.3 ± 16.8	23.5 ± 3.3	23.9 ± 3.2	6/63/21/0
Kocatürk et al. (25)	2019	Turkey	Prospective	Pleural biopsies	293	55.1 ± 17.2	52.2 ± 15.7			21.4/44.1/29 /5.5
Lan et al. (26)	2018	China	Retrospective	Lobectomy	119	55.34 ± 13.83	56.98 ± 11.05	22.40 ± 2.85	22.51 ± 2.57	82.4/16.8/0.8/0
Liang et al. (27)	2019	China	Retrospective	Mediastinal tumor resection	198	45.61 ± 14.08	48.48 ± 14.64	22.93 ± 2.58	23.2 ± 3.62	8/91/1/0
Liu et al. (28)	2021	Taiwan	Retrospective	Segmentectomy	86	60.5 ± 12.1	58.2 ± 13.0	22.1 ± 2.0	22.4 ± 2.7	16.3/58.1/23.3 /2.3
Liu et al. (29)	2014	China	RCT	Bullectomy	354	32.7	28.7			
Liu et al. (29)	2014	China	RCT	Lobectomy	356	56.2	56.2			
Liu et al. (29)	2014	China	RCT	Wedge resections	355	55.7	50.6			
Liu et al. (30)	2016	China	Retrospective	Lobectomy	339	56.0 ± 10.3	57.3 ± 10.5	22.4 ± 2.5	22.5 ± 3.43	
Liu et al. (30)	2021	China	Retrospective	Segmentectomy	339	51.2 ± 13.0	56.0 ± 12.8	22.2 ± 2.2	22.4 ± 3.1	
Liu et al. (31)	2019	China	Retrospective	Mediastinal tumor resection	225	59.4 (33–67)	57.3 (37–76)	22.7 (17.1–33.5)	23.2 (16.6–31.0)	Only ASA I and II
Mao et al. (32)	2021	China	Retrospective	Mediastinal tumor resection	40	43.90 ± 15.18	54.26 ± 11.64	23.01 ± 3.64	23.49 ± 2.52	47.62/52.38
Metelmann et al. (33)	2021	Germany	Retrospective	Elective minor VATS procedure	104	55.43 ± 18.71	57.83 ± 18.12	25.13 ± 4,565	26.37 ± 4.38	13.04/47.82 /34.78/4.35
Mineo et al. (34)	2014	Italy	Retrospective	Pleural effusions	231	66.0 ± 10.5	64.7 ± 12.7			
Pompeo et al. (9)	2012	Italy	RCT	Lung resections	63	64 ± 9	65 ± 7	24 ± 4	23 ± 3	No ASA IV
Pompeo et al. (7)	2007	Italy	Retrospective	Pneumothorax	49	28 ± 14	26 ± 11			
Wang et al. (35)	2021	Taiwan	Retrospective	Lobectomy	194	59.6 ± 11.3	61.9 ± 11.5	103.9 ± 7.1	114.1 ± 6.4	
Akopov et al. (36)	2015	Russia	Prospective trial	Lung abscess	65	58.4 (24 to 78)				0/5/29/66
Ambrogi et al. (37)	2014	Italy	Cohort study	Wedge resection	20	57 (36–76)		26.2 (17–38)		
Chen et al. (38)	2016	China	Cohort study	Endoscopic thoracic sympathectomy	58	24.3 (17–48)				
Chen et al. (39)	2016	China	Cohort study	Endoscopic thoracic sympathectomy	85	23 (16–45)				
Chen et al. (40)	2014	Taiwan	Retrospective	Metastasectomy	446	56.9 ± 16.8				
Cherchi et al. (41)	2020	Italy	Retrospective	Lung biopsies	97	66 ± 10		27.0 ± 4.7		
Hung et al. (42)	2015	Taiwan	Retrospective	Lobectomy	238	59.7 ± 11.4		22.9 ± 2.6		11.8/61.7/26.5 /0
Hung et al. (43)	2014	Taiwan	Retrospective	Lung nodules	32	52.8 ± 11.3		22.0 ± 2.4		41/50/9/0
Hung et al. (44)	2014	Taiwan	Retrospective	Mediastinal or pleural tumors	109	56.4 ± 14.0		22.3 ± 2.8		19.3/64.2/16.5 /0
Hung et al. (45)	2013	Taiwan	Retrospective	Lobectomy	21	61.0 ± 15.2		22.8 ± 3.6		9.5/52.4/38.1/0
Hung et al. (46)	2019	Taiwan	Retrospective	Lobectomy	1,025	59.3 ± 12.3		22.6 ± 2.7		
Li et al. (47)	2020	China	Prospective trial	Lung resections or sympathectomy	57	42.3 ± 19.5		<28 kg/m^2^		Only ASA I and II
Liu et al. (48)	2018	Taiwan	Retrospective	Sublobar resection	50	53.6 ± 18.0				
Liu et al. (49)	2020	Taiwan	Retrospective	Segmentectomy	32	58.3 ± 13.2		21.9 ± 2.0		21.9/59.4/15.6 /3.1
Liu et al. (50)	2020	Taiwan	Retrospective	Wedge resection	55	44.8 ± 11.1				89/11/0/0
Moon et al. (51)	2018	Korea	Retrospective	Lung resections, mediastinal or pleural tumors	115	61.8 (± 13.3)		23.8 (± 3.0)		
Pompeo et al. (2)	2019	Italy	Retrospective	Lung biopsies	112	60 ± 12		26 ± 3		
Pompeo et al. (8)	2011	Italy	Retrospective	Bullectomy	35	60 (55–65)		23.9 (22–27)		
Starke et al. (52)	2020	Germany	Retrospective	Minor thoracic surgery	88	60.14 ± 17.42		25.94 ± 4.95		10.4/33.3/47.9 /8.3
Starke et al. (52)	2020	Germany	Retrospective	Major thoracic surgery	89	67.94 ± 12.28		24.05 ± 4.42		0/5/75/0
Tseng et al. (53)	2012	Taiwan	Retrospective	Lung nodules	46	54.5 ± 11.5				8.7/76.1/15.2/0
Wang et al. (54)	2017	Taiwan	Retrospective	Lung resections	188	56.0 ± 12.5		22.7 ± 3.3		20.2/58.0/21.8 /0
Wang et al. (55)	2018	Taiwan	Retrospective	Lung resections	28	68.8 ± 12.8		22.0 ± 2.3		0/18/79/4

**Figure 2:**
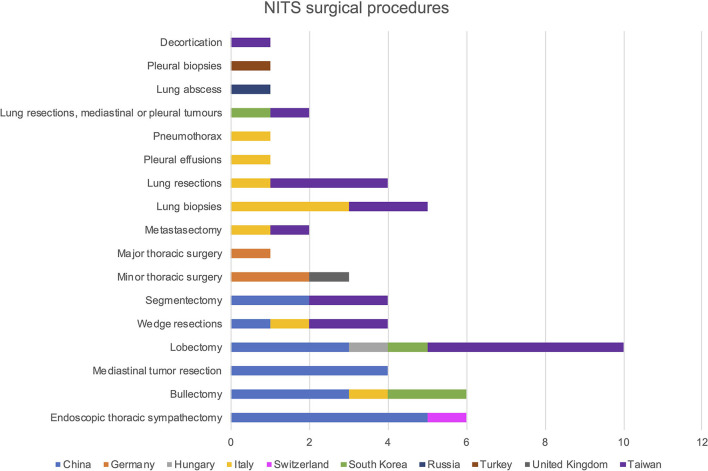
Frequency distribution of NITS surgical procedures by country of origin.

Regarding the described surgical procedures: 17.9% were lobectomies, 10.7% endoscopic thoracic sympathectomies, 10.7% bullectomies, 8.9% lung biopsies, 7.1% mediastinal tumor resections, wedge resections, segmentectomies, and lung resections, respectively. In 5.4% of cases the procedure was classified as minor thoracic surgery, without reporting a precise surgical technique, 3.6% were mastectomy and lung resections, mediastinal or pleural tumors, and major thoracic surgeries. Pleural effusions, pneumothorax, lung abscess, pleural biopsies, and decortications were reported in the 1.8% of cases. Surgical procedures as used in different Country are reported in Figure 2.

#### Anesthesiological and Intraoperative Management

**Figure 3:**
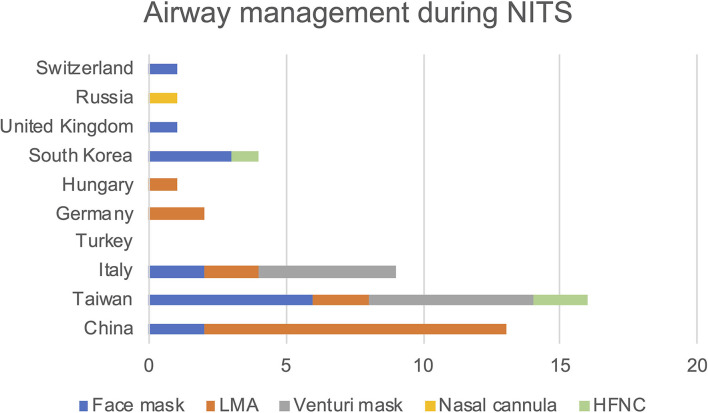
Frequency of airway management devices used within the various clinical trials divided by country of the primary country in which the study was conducted.

Not all studies reported information on how the airways were managed. Laryngeal mask (LMA) was used in 37.5% of cases, facemask in 31.3% of cases, Venturi mask in 22.9% of cases, high-flow nasal cannulas (HFNC) in 6.3%, and nasal cannulas in 2.0%. The type of device used in different Countries is reported in Figure 3.

Average conversion rate to intubation was 0.9% in China, 3.9% in Taiwan, 2.3% in Italy, 1% in Turkey, 5% in Germany and 3.7% in South Korea. The conversion rate to thoracotomy was estimated to be 0.1% in China, 0.3% in Taiwan, 2.3% in Italy, and 3.5% in South Korea. Not all studies reported these data.

#### Main Outocomes

Length of hospital stay and postoperative pulmonary complications rate, as collected in studies comparing the cohort of patients treated with NITS and intubated VATS, are shown in Table 2.

**Table 2:** Summary table of population characteristics of cohort studies.

**Author**	**Country**	**Type of study**	**Surgical procedure**	**N of patients**	**Postoperative pulmonary complications group A vs. group B**	**LOS group A vs. group B**
AlGhamdi et al. (10)	Korea	Retrospective	Lobectomy	62	Not significant	Not significant
Ambrogi et al. (11)	Italy	Retrospective	Metastasectomy	58		Significant, favors NITS
Caviezel et al. (12)	Switzerland	Retrospective	Endoscopic thoracic sympathectomy	20	Not significant	
Chen et al. (13)	Taiwan	Retrospective	Lobectomy	30	Not significant	Significant, favors NITS
Chen et al. (14)	China	RCT	Endoscopic thoracic sympathectomy	168	Not significant	
Chen et al. (15)	China	RCT	Endoscopic thoracic sympathectomy	221	Not significant	
Cui et al. (3)	China	Retrospective	Bullectomy	90	Not significant	Significant, favors NITS
Cui et al. (3)			Endoscopic thoracic sympathectomy	89	Not significant	Significant, favors NITS
Cui et al. (3)			Mediastinal tumor resection	91	Not significant	Significant, favors NITS
Furák et al. (16)	Hungary	Retrospective	Lobectomy	38	Significant, favors NITS	
Guerrera et al. (6)	Italy	Prospective	Lung biopsies	94	Significant, favors NITS	Significant, favors NITS
Guo et al. (17)	China	Retrospective	Segmentectomy	140	Not significant	Significant, favors NITS
Guo et al. (18)	China	Retrospective	Bilateral bullectomy	37	Not significant	Not significant
Hsiao et al. (19)	Taiwan	Retrospective	Decortication	33	Significant, favors NITS	Significant, favors NITS
Huang et al. (20)	China	Retrospective	Mediastinal tumor resection	32	Not significant	Not significant
Hwang et al. (21)	Korea	Retrospective	Bullectomy	41	Not significant	Significant, favors NITS
Irons et al. (22)	United Kingdom	Retrospective	Elective minor VATS procedure	73		Not significant
Jung et al. (23)	Korea	Retrospective	Bullectomy	183		Significant, favors NITS
Ke et al. (24)	Taiwan	Retrospective	Lung resections	160	Not significant	Significant, favors NITS
Kocatürk et al. (25)	Turkey	Prospective	Pleural biopsies	293	Not significant	Significant, favors NITS
Lan et al. (26)	China	Retrospective	Lobectomy	119		Significant, favors NITS
Liang et al. (27)	China	Retrospective	Mediastinal tumor resection	198		Significant, favors NITS
Liu et al. (28)	Taiwan	Retrospective	Segmentectomy	86	Not significant	Not significant
Liu et al. (29)	China	RCT	Bullectomy	354	Significant, favors NITS	Significant, favors NITS
Liu et al. (29)			Lobectomy	356		Significant, favors NITS
Liu et al. (29)			Wedge resections	355		Not significant
Liu et al. (30)	China	Retrospective	Lobectomy	340	Not significant	
Liu et al. (30)			Segmentectomy	339	Not significant	Significant, favors NITS
Liu et al. (31)	China	Retrospective	Mediastinal tumor resection	225	Not significant	Not significant
Mao et al. (32)	China	Retrospective	Mediastinal tumor resection	40		Not significant
Metelmann et al. (33)	Germany	Retrospective	Elective minor VATS procedure	104	Not significant	Not significant
Mineo et al. (34)	Italy	Retrospective	Pleural effusions	231	Not significant	Not significant
Pompeo et al. (9)	Italy	RCT	Lung resections	63		Significant, favors NITS
Pompeo et al. (7)	Italy	Retrospective	Pneumothorax	49		Significant, favors NITS
Wang et al. (35)	Taiwan	Retrospective	Lobectomy	194		Not significant

**Figure 4:**
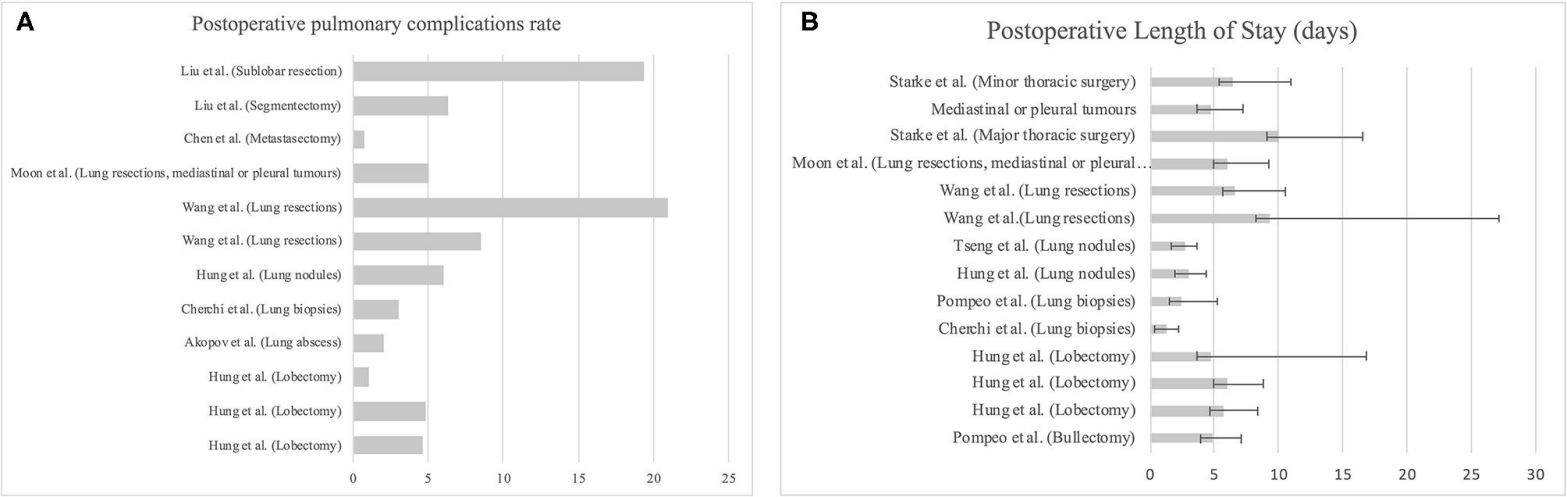
**(A)** Postoperative pulmonary complications expressed as frequency split by type of surgery. **(B)**. Length of stay, expressed as mean and standard deviation, split by type of surgery.

Same data as collected from single cohort studies are instead shown in Figure 4. In Figure 4 are also presented the data relating to pulmonary complications by type of procedure as taken from single cohort studies.

#### Perioperative Analgesia

Analgesia performed in the perioperative period was not reported in all studies. Of the studies analyzed, 42.4% used intercostal nerve block, 40.7% thoracic epidural analgesia, 6.8% paravertebral block, 5.1% infiltration with local anesthetic of the wound, 3.4% erector spinae plane block, and 1.6% placed a catheter at the paravertebral site for continuous analgesia. Evaluating NITS vs. non-NITS cohort studies the postoperative pain was significantly lower in the NITS group in five studies (3, 6, 14, 15, 22), whereas the findings were not statistically significant in six studies (19, 21, 25, 28, 31, 32).

**Figure 5:**
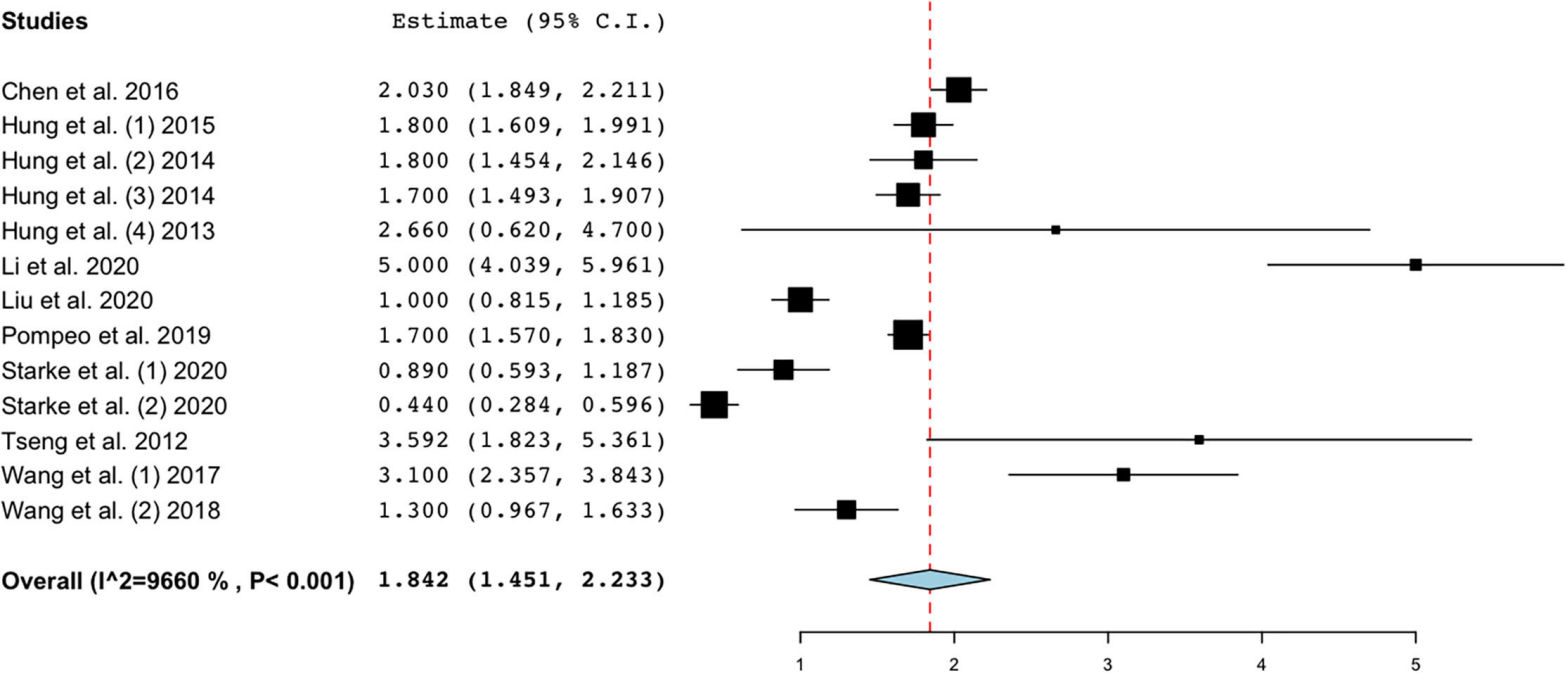
Postoperative pain on day 1 expressed in VAS. Evaluation by all observational studies. Figure created with OpenMetaAnalyst.

Meta-analyzing the available data from observational studies (Figure 5) with a continuous random-effects model showed that the mean postoperative pain rate (VAS) is 1.842 (95% C.I. 1.451–2.233) with a heterogeneity of 96.6%, *p* < 0.001.

## Discussion

In this scoping review, we analyzed 53 primary studies regarding NITS published between 2011 and 2021. The studies are all fairly recent confirming that NITS procedures have gained acceptance quite recently.

One of the objectives of this analysis was to evaluate the clinical context in which NITS is performed. Since the consensus of experts we referred to did not investigate this particular aspect in detail (4), we decided to assess whether there is a type of surgical procedure or airway management on which there is consensus among different Countries. What seems to emerge is that NITS is a procedure performed predominantly in Asia, and in some European countries, first Italy. We did not find any studies conducted in the United States. The trials conducted are mostly focused on selected populations, allowing direct comparison between intubated and non-intubated thoracic surgery. The shortage of clinical trials justifies the lack of consensus and guidelines on its management.

We evaluated the type of surgery performed during NITS by Country: while in China it was mainly used in lung parenchymal surgery, and in sympathectomies for hyperhidrosis, in Italy, it was mainly adopted for minor thoracic operations, such as lung biopsies or pleural effusions, confirming what Pompeo et al. reported in their European survey (56). In Taiwan instead, NITS procedures were used in a more heterogeneous manner, including both major parenchymal procedures, such as lobectomies, and minor procedures.

When assessing the anthropometric characteristics of the population, we found that there was a considerable heterogeneity, and this was probably due to a lack of specific guidelines indicating the population in which NITS procedures is most correctly applied. Even considering the NITS expert consensus, we noted that only ASA I and II stage patients, aged between 16 and 60 years, were included. This, in our opinion, hardly represents the average patient who ordinarily undergoes thoracic surgery. Moreover, we believe that such procedures would be rather helpful in frail patients with ASA III and IV status to avoid the stress related to intubation and positive pressure mechanical ventilation in patients with compromised respiratory function (3). There are cases in literature where this technique is used in patients with severe comorbidities, including obesity (57, 58) whereas other groups considered them as contraindications (59).

Regarding airway management, we noted that there was a difference among the Countries considered. The facemask was the most widespread device across the board. In China the LMA was used in most cases, as well as in Germany and in Hungary. This can easily be related to the fact that these Countries mainly performed major thoracic surgery. Although the LMA support during NITS has been described in the literature, it certainly does not allow lighter sedation. To date, there is no recommendation on which device to preferentially use for airway management (60, 61) even if He et al. suggest the use of LMA, nasal cannulas, or face mask as alternatives (4). From our review, the type of device for airway management is highly dependent on the background of the study and the practices of individual centers.

In this regard, a fundamental issue on NITS definition arises, as three studies conducted in China used LMA with extemporaneous curarization, and 22 studies reported during surgery, BIS values below 60, as under general anesthesia. There is still a lack of definition on the depth of sedation in the context of NITS, and this has resulted in the development of other terminologies we refer to, for example, awake thoracic surgery. However, it is confusing and does not allow focusing on the NITS technique in a univocal way: the expert consensus should aim to provide more information on the depth of sedation in NITS contexts.

The rate of conversion to intubation was highly variable from Nation to Nation, as it was the rate of thoracotomy. Considering the percentages reported in these articles, the average conversion rate to orotracheal intubation was about 3%. The lower rate of intubation found in China could be related to its prevalent use of the LMA. It is worth noting the unexpectedly high conversion rate observed in Germany despite the diffuse use of LMA.

Referring to outcome, we found that in 83% of cases there were no significant differences between the two cohorts, whereas in 27% NITS was proven to reduce pulmonary complications. Also, regarding the effectiveness in limiting the LOS, in 63% of cases NITS was considered more effective while 37% found no statistically significant difference between the two groups (Table 2).

When evaluating single cohort studies, pulmonary complications predominantly developed after parenchymal surgery, whereas for the LOS we did not see a clear correlation with the type of surgical procedure (Figure 4). According to Lan et al. (26) patients who underwent NITS had a higher incidence of atelectasis, pleural effusion, or pulmonary exudation in the face of a better LOS and general postoperative comorbidities compared with intubated patients. From the perspective of NITS and pulmonary complications, this issue remains controversial. In four recent meta-analyses, it is confirmed that NITS would appear to reduce the LOS, providing further validation for our analysis (62–65).

From a pain perspective, our findings were inconclusive: there was no evidence to prove the superiority of NITS in terms of postoperative pain over intubated thoracic surgery. About half of the cases had nonsignificant postoperative pain between the two groups; no regional anesthesia was performed but only sedation. Therefore, this fact might have impacted the final result. In contrast, regional anesthesia had been performed in all studies in which there was a statistically significant difference between the two groups. When evaluating postoperative pain among observational studies, although there was high heterogeneity (I2 = 96.6%) a very low score of pain at postoperative day 1 was found (Figure 5). The NITS technique, accompanied by regional anesthesia, might be a good way to reduce postoperative pain in surgery at high risk of developing persistent postoperative pain and prone to high acute postoperative pain (66, 67). Reducing opioid consumption in commonly frail patients, such as those undergoing NITS, could affect postoperative hospitalization and complication outcomes (6).

This study has limitations. The major is that it is based on mostly retrospective studies. Results therefore should always consider the low quality due to bias from retrospective studies.

## Conclusion

NITS procedures are becoming increasingly popular, but they need more definition, especially the setting in which they are performed. It would be necessary, for example, to reach an agreement on the patient sedation, and airway management devices to perform NITS techniques in the same way across the countries. The choice of surgical procedure, as well as that of the patient, have not been well-defined in the literature yet. It is our opinion that frail patients have fewer complications during NITS than during intubated thoracic surgery (6). From a postoperative patient management perspective, the impact of NITS techniques on LOS remains unknown as the existing evidence available in the literature is conflicting. A regional anesthesia approach might be recommended in NITS procedures to reduce acute postoperative pain. Future studies should be directed to evaluate the benefits of NITS in patients with impaired lung function or other comorbidities (e.g., obesity, ASA III, ASA IV). Moreover, other randomized controlled trials are needed to establish more robust evidence.

## Data Availability Statement

The raw data supporting the conclusions of this article will be made available by the authors, without undue reservation.

## Author Contributions

GR: conceptualization, formal analysis, investigation, methodology, project administration, supervision, validation, visualization, writing—original draft, and writing—review & editing. PL: data curation, software, visualization, writing—original draft, and writing—review & editing. EC: conceptualization, formal analysis, investigation, methodology, project administration, validation, writing—original draft, and writing—review & editing. EB and MC: data curation, formal analysis, investigation, visualization, and writing—original draft. FP: writing—original draft and writing—review & editing. ER: validiation and writing—review & editing. LB: conceptualization and writing—review & editing. FG: conceptualization, project administration, resources, methodology, supervision, validation, writing—original draft, and writing—review & editing. All authors contributed to the article and approved the submitted version.

## Conflict of Interest

The authors declare that the research was conducted in the absence of any commercial or financial relationships that could be construed as a potential conflict of interest.

## Publisher's Note

All claims expressed in this article are solely those of the authors and do not necessarily represent those of their affiliated organizations, or those of the publisher, the editors and the reviewers. Any product that may be evaluated in this article, or claim that may be made by its manufacturer, is not guaranteed or endorsed by the publisher.
